# The Impact of Adverse News About the Gaza War on the Health of Iranian Elderly People: A Qualitative Study

**DOI:** 10.1002/hsr2.71762

**Published:** 2026-01-28

**Authors:** Milad Ahmadi Marzaleh, Katayoun Jalali, Mahmoudreza Peyravi, Mohammad Hasan Keshavarzi, Rita Rezaee, Mostafa Moazam Fard

**Affiliations:** ^1^ Department of Health in Disasters and Emergencies, School of Health Management and Information Sciences, Health Human Resources Research Center Shiraz University of Medical Sciences Shiraz Iran; ^2^ Student Research Committee Shiraz University of Medical Sciences Shiraz Iran; ^3^ Clinical Education Research Center, School of Medicine Shiraz University of Medical Sciences Shiraz Iran

**Keywords:** elderly, media, mental health, physical health, war

## Abstract

**Introduction:**

Studies on the responses of older people who have experienced disasters are contradictory, suggesting two opposing theoretical approaches: the vulnerability and resilience approaches. Therefore, this qualitative study examines the impact of the media on Iranian elderly people during the Gaza war in 2024.

**Methods:**

This qualitative study employed purposive sampling to select 12 elderly participants aged 65 years and older. Data collection was done from September to December 2024 through semi‐structured interviews. The interviews were transcribed, and the data analysis was conducted following the steps outlined by Graneheim and Lundman.

**Results:**

Results revealed three major themes: emotional exhaustion, physical stress responses, and resilience through social connection. Participants reported symptoms such as sleep disturbances, elevated blood pressure, and persistent anxiety after prolonged exposure to war‐related media. Despite these challenges, some individuals demonstrated adaptive coping strategies, including limiting media intake and engaging in community support programs. These findings highlight the dual impact of media exposure—both distressing and mobilizing—among elderly populations.

**Conclusion:**

It is necessary to strengthen the elderly's resilience to reduce the psychological and physical stress effects of war news on them. This can be achieved by practicing stress control and management, emphasizing social connections, developing a proper understanding of the media environment, and, most importantly, monitoring the contents broadcast by the media. Implementing a violent content label to give the audience the right to choose can help prevent media‐related risks. These measures are crucial for this purpose.

## 1 Introduction

War is one of the greatest threats to the health and well‐being of societies. Indeed, war affects everyone and does not discriminate against any side. Individual and societal health are the immediate victims of war. However, the interconnectedness of today's world means that the impact of war is felt thousands of miles away, even by people who are not directly involved in the conflict [1].

Extensive media coverage extends the boundaries of local disasters, spreading their impact far beyond the populations directly exposed and transforming them into mass traumas with potentially harmful health effects. For example, exposure to television news during the Oklahoma City bombing, the 1990 Gulf War, and similar events has been associated with a widespread occurrence of trauma symptoms immediately following the incidents. Media exposure after 9/11 has even been prospectively associated with increased post‐traumatic stress symptoms over the 3 years following the attacks [2]. In today's world, social media is one of the most widely used sources of information globally. Affordable access to the Internet, easy entry, and a significant number of users have made social media one of the easiest and most effective ways to disseminate information [3]. Operating mostly within pre‐existing frameworks, the media combine dominant sociopolitical assumptions with current information to create narratives that are likely to influence the audience in predictable ways [4, 5, 6, 7].

Overall, social media allows older adults to express themselves, participate in discussions, and stay connected with their community [8]. Participation in social networks can empower older adults and provide them with a greater sense of connection, control, and self‐efficacy [9]. The elderly constitute the most rapidly growing population in the world [10], and their social needs can be met through participation in social networks [11, 12]. Social networks have become a vital part of everyday life for many people. In recent years, an increasing number of older people have started to use social media [13, 14, 15, 16].

In a recent regional study, Al‐Maghaireh and colleagues examined acute stress disorders among Jordanian adolescents after viewing Gaza war footage on social media. Their findings revealed heightened anxiety, emotional dysregulation, and trauma‐like symptoms, underscoring the psychological impact of indirect conflict exposure in neighboring populations [17].

While the trauma of war affects everyone, the elderly are one of the most vulnerable groups to both immediate and long‐term consequences [1]. In addition, the World Health Organization recognizes older people as a vulnerable group in emergencies [18]. Studies on the responses of older adults who have experienced disasters have been contradictory, suggesting two opposing theoretical approaches [19]: vulnerability perspectives versus resilience perspectives [20, 21, 22]. The vulnerability perspective suggests that exposure to severe stress increases older adults' susceptibility to emotional problems and reduces their ability to withstand additional stress. On the other hand, the resilience perspective suggests that coping capacity increases with age. It hypothesizes that exposure to stress enhances individuals' resistance to subsequent stress and ultimately protects them from harm [23]. The importance of promoting a culture of resilience and supporting community resilience to disasters—that is, the ability of communities to withstand, respond to, and recover from extreme events—is increasingly recognized [18]. However, older people are considered more vulnerable when it comes to coping with post‐traumatic stress symptoms and recovering from the shock of war [23]. Despite extensive international research on media‐induced trauma and stress, particularly among younger populations and direct conflict survivors, there remains a significant gap in understanding how elderly individuals in noncombat regions psychologically respond to prolonged exposure to war‐related media. Most existing studies focus on adolescents, refugees, or frontline communities, often overlooking older adults who may experience indirect trauma through continuous media engagement. Furthermore, few studies have explored this phenomenon within culturally and geographically proximate contexts to the Gaza conflict, such as Iran. This study addresses that gap by examining the emotional and physical responses of elderly Iranian individuals exposed to Gaza war coverage, offering new insights into vulnerability and resilience mechanisms in this neglected population.

## 2 Methods

In‐depth, semi‐structured interviews were conducted in Persian over 4 months from September to December, with approval from the university Vice Chancellor for Research. Each participant was interviewed individually, and the subjects' experiences were extracted in alignment with the study objectives. The participants were asked to recall their experiences related to the effects of exposure to news about the Gaza war. The time and location of the interviews (in a private room) were arranged with participants who consented to participate. The interview questions included: “What effects have the media reports of the Gaza war had on you?” and “What were the psychological, emotional, and physical effects?” Based on participants' responses, follow‐up questions, such as “How? Why? Can you explain more?”, were also posed. The interviews ranged from 30 to 90 min and were recorded with the participants' permission. Data collection concluded when data saturation was reached.

This study adopts an interpretivist epistemological stance, grounded in the belief that reality is socially constructed and best understood through the subjective experiences of individuals. Given the focus on elderly participants' emotional and physical responses to war‐related media, a qualitative design using conventional content analysis was deemed appropriate. This framework allows for systematic identification and categorization of the themes which emerged from participants' narratives, shaped by cultural, historical, and personal contexts. The research method—semi‐structured interviews and content analysis following Graneheim and Lundman's [24] approach—was selected to align with this interpretivist orientation, emphasizing depth, nuance, and contextual sensitivity.

### 2.1 Participants

The study was conducted in Shiraz, a culturally rich city in southern Iran known for its historical emphasis on poetry, spirituality, and community cohesion. The elderly population in this region often maintains strong familial ties and engages actively with televised and social media content, especially regarding regional conflicts. Cultural values such as empathy toward oppressed populations and collective mourning practices may intensify emotional responses to war‐related media. These cultural dynamics provide a meaningful context for interpreting participants' psychological and physical reactions to Gaza war coverage.

The statistical population of this study consisted of the elderly over the age of 60 who had been continuously exposed to news of the Gaza war from the media for about 6 months. Exclusion criteria included participants who refused to continue cooperating. Participants were recruited through purposive sampling from community health centers and local elderly associations in Shiraz. The sampling strategy followed a criterion‐based approach, targeting individuals aged 60 and above who reported regular exposure to televised or social media coverage of the Gaza war. Maximum variation was considered in terms of gender, education level, and prior war‐related experiences. Continuous exposure was assessed through self‐report during initial screening, where participants confirmed daily or near‐daily engagement with war‐related news since April 2024. A total of 12 elderly people were selected and interviewed.

Inclusion criteria were as follows: (1) individuals aged 60 years or older, (2) residence in Shiraz for at least the past 12 months, (3) self‐reported continuous exposure to televised or social media coverage of the Gaza war for a minimum of 6 months, (4) ability to participate in a semi‐structured interview, and (5) lack of diagnosed cognitive impairment or severe psychiatric conditions that would interfere with informed consent or data collection.

Media exposure was operationalized as daily or near‐daily engagement with televised or social media coverage of the Gaza war for a minimum of 6 consecutive months. During initial screening, the participants self‐reported their exposure patterns, including frequency, preferred media sources, and average daily duration. Follow‐up questions were used to confirm consistency of exposure. Only individuals who reported continuous engagement over the 6 months were included in the study.

### 2.2 Data Collection and Data Analysis

Semi‐structured interviews were conducted to explore the elderly participants' emotional and physical responses to war‐related media. The interview guide consisted of broad, open‐ended questions designed to elicit participants' narratives in their own words. To ensure adequate depth, we consistently used probing questions (e.g., “How?”, “Why?”, “Can you explain more?”) to encourage elaboration and clarify meanings.

Reflexivity was addressed throughout the study. The lead researcher maintained a reflexive journal to document personal assumptions, emotional reactions, and potential influences on the interview process. Peer debriefing was conducted with academic colleagues to minimize interviewer bias and enhance credibility. Member checking was also employed, allowing the participants to confirm the accuracy of preliminary interpretations. These procedures ensured methodological rigor and transparency in the data collection process.

Data analysis was conducted simultaneously with data collection using the five‐step content analysis approach proposed by Graneheim and Lundman [24] (Table 1).

1. The transcripts of each interview were transcribed verbatim and carefully read several times to gain a general understanding of the content.
2. Sentences relevant to the research topic were identified as units of meaning.
3. Initial codes were extracted.
4. The extracted codes were classified into conceptual categories based on similarities and differences.
5. Further abstract concepts were then generated by systematically comparing the initial conceptual categories.

**Table 1:** Demographic characteristics of the study participants.

Characteristics	Divisions	Frequency (%)
Gender	Male	8 (66%)
	Female	4 (34%)
Relationship status	Married	12 (100%)
	Single	0
Education level	Diploma	3 (25%)
	Bachelor's degree	5 (41.6%)
	Master's degree	3 (25%)
	PhD	1 (8.4%)

Meaning units relevant to the research question were identified and condensed; then, the initial codes were assigned. Codes were systematically compared and grouped into categories based on similarities and differences. Categories were further abstracted into themes. A coding matrix was constructed to illustrate the progression from raw data to codes, categories, and themes. Exemplary initial codes and representative quotations from participants were included to demonstrate how the themes were grounded in the narratives. Thematic refinement was conducted collaboratively by the research team to minimize overlap and ensure clear differentiation between the themes and subthemes.

Data saturation was assessed iteratively during the data collection and analysis process. Saturation was achieved after ten interviews. After the tenth interview, the research team conducted a preliminary thematic review and found that no new codes or concepts were emerging. Two additional interviews were conducted to confirm this pattern. Saturation was determined collaboratively by the leading researcher and two peer reviewers, based on the repetition of the themes and the absence of novel insights. Although the sample size was 12, the participants represented diverse backgrounds in terms of gender, education, and war‐related experiences, which contributed to thematic richness. The decision to stop at 12 was guided by the principle of informational redundancy rather than numerical adequacy.

Data saturation was evaluated in an iterative manner during both data collection and analysis. The determination of saturation was made collaboratively by the lead researcher and two peer reviewers, based on the repeated emergence of themes and the absence of novel insights. After the tenth interview, a preliminary thematic review was conducted by the leading researcher and two peer reviewers. They found that no new codes or concepts were emerging, and existing themes were consistently repeated. Two additional interviews were conducted to confirm this pattern. Saturation was determined collaboratively based on informational redundancy and thematic repetition.

The analysis was conducted primarily by the first author, with continuous supervision by the other authors. One observer analyzed parts of the data alongside the first author, and interpretations were then continuously discussed with all authors at all stages, including coding, grouping of codes, and levels of interpretation of themes, to ensure reliability.

### 2.3 Trustworthiness and Rigor

For assessment of the trustworthiness of the collected data, we used Guba and Lincoln's standards for scientific rigor in qualitative research: credibility, confirmability, dependability, and transferability [25]. To enhance credibility and dependability, the researcher continuously engaged with the data and reviewed the interview texts multiple times for a comprehensive understanding. This process was further strengthened by ensuring maximum diversity in sampling, member checking, peer checking, and external debriefing. In peer review, two qualified researchers assessed the accuracy of the data analysis. During member checking, some participants confirmed the alignment between the study findings and their experiences. For transferability, clear explanations were provided about various aspects of the study, including sampling, data collection, and the research field. To ensure dependability, we preserved all documents related to the study so that others could verify the study process.

Member checking was conducted with 5 out of the 12 participants, selected to reflect diversity in gender, education, and emotional response. Each participant received a brief written summary of the preliminary findings via phone messaging or in‐person follow‐up and was asked to confirm whether the interpretations aligned with their experiences. Feedback was incorporated into the final thematic structure. Peer checking was conducted by two academic colleagues with doctoral‐level training in qualitative research and public health. They independently reviewed the coding framework and thematic analysis to ensure consistency, credibility, and alignment with the data.

### 2.4 Ethical Considerations

All ethical considerations were thoroughly addressed, and the study protocol was reviewed and approved by the Ethics Committee of Shiraz University of Medical Sciences (IR.SUMS.NUMIMG.REC.1404.024). After securing the necessary permits from the Vice Chancellor of Research, the Faculty of Medical Management and Informatics and obtaining a letter of approval from Shiraz University of Medical Sciences, the researchers introduced themselves and explained the study objectives, along with the research samples. Participants were assured that all recorded information would remain confidential.

To safeguard the participants' emotional well‐being during potentially distressing interviews, we implemented several protective measures. Interviews were conducted in a quiet, private setting, and participants were informed in advance about the nature of the questions. They were reminded of their right to pause or withdraw at any time with no consequence. The interviewer maintained a supportive and nonjudgmental tone throughout, and emotional cues such as distress or discomfort were closely monitored. In cases where participants showed signs of emotional fatigue, interviews were gently shortened or rescheduled. These steps were taken to ensure psychological safety and uphold ethical standards in line with the Declaration of Helsinki.

Subsequently, individuals who expressed their willingness to participate in the study were selected, and they were further reassured that they could withdraw at any stage of the interview process. Additionally, the Declaration of Helsinki was considered in this study. Additional ethical measures included:

1. Providing participants with comprehensive information about the study.
2. Obtaining written informed consent from elderly participants.
3. Expressing gratitude and acknowledgment to all participants.
4. Respecting each participant's freedom to continue or withdraw from the interviews or the study at any time.

## 3 Results

The majority of the participants were male (*N* = 8), with a mean and SD of age of 68 ± 12 years. The demographic characteristics of the participants are presented in Table 1.

Data analysis of the factors influencing the adverse impact of news about the Gaza war on the health of Iranian elderly individuals identified 4 main themes and 18 subthemes. The primary categories were psychological effects, physical stress, connection to memories, and engagement in supportive activities (Table 2).

**Table 2:** The main theme and subthemes extracted from the interviews.

Theme	Subthemes
Psychological impact	Depression
	Restlessness
	Anger
	Anxiety
	Aggression
	Lack of concentration
	Fear
	Stress
	Sadness
Physical tension	Headache
	Heart palpitations
	Hand tremors
	Increased blood pressure
	Shortness of breath
	Stomach pain
Recalling historical memories	Remembering bad memories of the past
	Mental rumination
Engagement in advocacy activism	Support for victims

The first theme identified through data analysis was psychological effects, encompassing subthemes such as depression, restlessness, anger, anxiety, aggression, lack of concentration, fear, stress, sadness, and grief. Hearing unfavorable news about the war triggered fear, stress, restlessness, and difficulty concentrating among elderly individuals, many of whom experienced feelings of depression and sadness:

A 67‐year‐old woman (P1) shared her experience: “If I were to elaborate on my discomfort, I would explain that hearing such news negatively impacted my concentration and daily mental tasks. For instance, if I hear distressing news, like the Gaza war and the massacre, while driving, I may lose focus and struggle to concentrate due to the severity of my discomfort. In such moments, I turn off the radio (an attempt to escape reality) or ask someone else to drive. I truly lack the concentration and balance required to perform my mental tasks.”

A 63‐year‐old man (P9) shared his experience, stating: “I frequently suffer from insomnia and inadequate sleep when I'm upset. When I dwell on these issues, I go to bed overwhelmed by fear and worry, often waking up with anxiety, bad dreams, and nightmares. These challenges lead to both physical and mental imbalances, and the adverse effects of such news hinder my ability to make decisive decisions.”

The second theme identified was physical stress, which included subthemes such as headaches, heart palpitations, hand tremors, increased blood pressure, shortness of breath, and stomach pain. Elderly individuals reported experiencing increased blood pressure, headaches, heart palpitations, and other physical symptoms of stress when exposed to news of war:

A 66‐year‐old man (P4) shared his experience, stating: “Sometimes, when I hear such news, my hands begin to shake, making it difficult even to hold a cup of tea. While my condition has improved with medication, hearing negative news or distressing reports, such as war in the media, triggers stress and heart palpitations again. Unfortunately, the situation often escalates to the point where I experience nightmares. The following day, due to the lack of sleep, I feel disoriented and unable to maintain the necessary balance at work. I end up needing to consult a doctor once more to address the issue.”

A 66‐year‐old woman (P11) stated: “When I feel upset, I struggle with a lack of focus on my work and find it impossible to complete tasks that require concentration. Seeing images of death and violence evokes anxiety, worry, and fear, along with a sensation of emptiness in my heart (as if it suddenly bursts). If the images are particularly distressing or we discuss them extensively at home or with friends, I experience nightmares and frequent urination due to fear and stress. Additionally, unlike many people, I tend to overeat when I'm upset, often consuming large amounts of food.”

A 64‐year‐old man (P8) stated: “Everyone gets upset and nervous at times, but for me, it triggers headaches and increases my blood pressure, requiring me to take medication. When I'm at home and come across this type of news, I feel even more distressed. For instance, when the Gaza hospital was attacked, I experienced a rapid heartbeat and felt so overwhelmed with stress and anxiety that I had to go to the hospital, where I was admitted overnight.”

The third theme highlights the connection to memories, with subthemes of recalling negative experiences from the past and engaging in rumination. When Iranian elderly individuals heard news of the war, they were reminded of the Iraq war and repeatedly revisited those memories and past events in their minds.

A 73‐year‐old man (P6) said: “When I hear news about the war, it lingers in my mind for a long time, and I cannot shake off these thoughts. This is because I witnessed how, during the war, our people constantly lived in stress and anxiety.”

The fourth theme reflected a desire to engage in advocacy activities. The elderly expressed a strong willingness to assist war victims in some capacity:

A 62‐year‐old man (P7) shared his experience as follows: *“*As an old individual, I know I need to avoid stress, but it's beyond my control. When I see images of war or hear related news, I become extremely stressed and lose focus. My mind gets so preoccupied that I end up reading a sentence multiple times without understanding it. I keep asking myself questions like: How are they coping now? Why isn't anyone helping them? I wish I could do something for them. These thoughts have occupied my mind for several days. Sometimes, I get headaches, and on one occasion, I even had to hit my head to calm myself and get a good night's sleep.”

## 4 Discussion

The findings of this study revealed that the impact of adverse news regarding the Gaza war on the health of Iranian elderly individuals could be categorized into four main themes: psychological effects, physical stress, connection to memories, and willingness to engage in supportive activities. These findings may reflect underlying psychological mechanisms such as vicarious trauma and media‐induced stress, where repeated exposure to violent imagery triggers emotional dysregulation, cognitive rumination, and somatic symptoms—even in individuals not directly involved in the conflict. To support these results, we reviewed similar studies in this field across various databases.

Recent studies have further emphasized the psychological impact of media exposure on older adults, particularly during crises. For instance, Bauldry and Stainback found that frequent consumption of fear‐inducing news during the COVID‐19 pandemic significantly increased psychological distress among elderly individuals in the United States, highlighting the role of media‐induced stress [26]. Similarly, Ragnhildsløkken and colleagues reported that social media use among older adults was associated with elevated anxiety and emotional fatigue, especially when exposed to negative news cycles MDPI [27]. These findings align with the current study themes of psychological and physical stress, suggesting that indirect exposure to conflict through media can trigger trauma‐like responses even in noncombat populations.

In terms of resilience, recent literature has explored coping strategies among older adults facing conflict‐related media exposure. While some individuals demonstrate adaptive behaviors—such as selective media avoidance or increased social engagement—others experience heightened vulnerability due to prior trauma or limited emotional support. The present study reflects both perspectives: some participants exhibited signs of emotional exhaustion and rumination (supporting the vulnerability model), while others expressed a desire to help and maintain social connection, indicating resilient coping mechanisms. This duality reinforces the need for nuanced interpretation within the vulnerability–resilience framework and suggests that media exposure may amplify pre‐existing psychological tendencies in elderly populations.

War is one of the greatest threats to the health and well‐being of societies, with its impact often persisting throughout life and beyond. Individual and societal health are among the immediate victims of war, and the consequences are often catastrophic for affected populations. One of the most significant outcomes of war is its devastating effect on people's mental health [1]. A 2022 study conducted across three countries—Poland, Ukraine, and Taiwan—compared mental health conditions, including depression, anxiety, stress, and post‐traumatic stress, as well as coping strategies and perspectives on the Russia–Ukraine war among the populations of these nations after the conflict began. The study also analyzed demographic, social, and economic factors associated with depression, anxiety, stress, and levels of post‐traumatic stress in all participants. The study results revealed that Ukrainian participants reported higher levels of depression, anxiety, stress, and post‐traumatic stress. Although Taiwanese participants were not directly involved in the war, their average mental health scores were slightly lower than those of Ukrainian participants. Taiwanese individuals reported significantly higher avoidance scores compared to Polish and Ukrainian participants. Additionally, more than half of the Taiwanese and Polish participants expressed distress when exposed to media war scenes [28]. In this study, one of the primary themes identified was the psychological impact of media news on older adults, as highlighted by many participants. This finding aligns with previous research, showing that repeated exposure to graphic media images of violence and death, particularly after terrorist attacks and wars, can result in psychological stress [2, 29].

Mass media can transform local disasters into national and global events, extending the negative impact of such incidents to a far wider audience than those directly affected. In these instances, events can evolve into mass trauma for many. For example, while tens of thousands of people directly witnessed the September 11 terrorist attacks, millions more experienced the event and its aftermath through media coverage. A 2012 study even found that September 11 had the most significant impact on television viewers in the past 50 years [29].

News coverage of major natural and man‐made disasters often captures significant public attention. Research on the impact of news coverage of natural disasters indicates that increased attention to media reporting on such events is correlated with heightened psychological distress among affected individuals [30]. While evidence indicates that direct exposure and video viewing of threatening content can induce fear states, the prevailing assumption in the field of professional mass trauma response is that individuals directly exposed to such events are at a high risk for stress‐related disorders. Nonetheless, exposure to a traumatic film can activate fear circuits in the brain, trigger flashbacks, and lead to post‐traumatic stress disorder [2].

According to a study by Silver and colleagues, spending 4 or more hours daily watching television and viewing images related to the September 11 attacks was linked to a higher incidence of physical and mental illnesses 2–3 years later. These findings indicate that exposure to media images can result in physical and psychological effects that were previously thought to require direct exposure to trauma [29]. In this study, one of the primary themes identified was physical stress resulting from repeated exposure to war news in the media. Many elderly participants reported experiencing physical stress alongside psychological stress. In this context, Kira and colleagues conducted a study entitled “Physical and Psychological Effects of Media Exposure to the Iraq War on Iraqi Refugees.” The study aimed to examine the impact of viewing or hearing news about the Iraq War on the physical and mental health of a sample of 501 Iraqi refugees. The findings revealed that exposure to war‐related news was a significant predictor of PTSD and health issues, even after considering the effects of prior cumulative trauma and demographic factors. The impact of media exposure on physical and mental health was comparable to experiencing the death or injury of family members and friends caused by war. The media holds significant power as it swiftly delivers potentially important and relevant information through vivid war imagery, which can negatively affect individuals [31]. Prior exposure to similar or violent events may render certain individuals more susceptible to the negative effects of mass trauma. Prolonged engagement with trauma‐related media content for several hours a day, particularly in the aftermath of mass trauma, can extend acute stress experiences and exacerbate stress‐related symptoms. Mass media may also act as a channel for spreading the adverse effects of social trauma beyond the directly impacted communities [2]. The psychological impact of war‐related media exposure, particularly among vulnerable populations such as the elderly, can be contextualized within international humanitarian and human rights frameworks. According to the Geneva Conventions and their Additional Protocols, civilians—especially older adults—are entitled to protection from the effects of armed conflict, including indirect harm. Furthermore, the United Nations' definition of psychological harm as a component of human suffering underscores the need to consider media‐induced distress as a legitimate area of concern. The findings of this study align with global calls to minimize civilian exposure to traumatic content and highlight the ethical responsibility of media outlets and policymakers to safeguard mental well‐being during times of conflict.

The study had limitations, including a lack of cooperation from some participants during interviews. To address this challenge, efforts were made to foster positive interactions with the participants. This study employed purposive sampling to recruit elderly individuals with sustained exposure to Gaza war news via the media. While data saturation was achieved after 12 interviews, the small sample size limits generalizability. Moreover, inclusion and exclusion criteria did not explicitly consider the participants' prior mental health conditions, trauma history, or direct war exposure, which may have influenced their psychological responses. Although the study aimed for maximum variation sampling in line with Guba and Lincoln's trustworthiness criteria, the final sample showed homogeneity in education level and marital status. This was largely due to contextual constraints in participants' availability and willingness to engage.

This study was conducted in a single city (Shiraz), which may limit the transferability of findings to other contexts. However, maximum variation sampling was applied to capture diverse perspectives within the city. The results should be interpreted within the cultural and social context of Shiraz, while offering insights that may be relevant to similar populations in other Iranian urban settings. Future research should consider broader geographic sampling, stratified recruitment, and screening for trauma‐related variables to enhance diversity and analytical depth.

Also, although credibility, dependability, and transferability were addressed through member checking and peer debriefing, confirmability could have been further strengthened. Formal strategies such as reflexive journaling or triangulation were not systematically applied, which may have limited the ability to fully minimize researcher bias during interpretation.

Moreover, the thematic structure may involve partial conceptual overlap, especially between psychological and memory‐related responses. While efforts were made to delineate the themes during analysis, future studies could benefit from applying theoretical frameworks (e.g., trauma theory or media psychology) to enhance interpretive clarity and depth.

All health‐related experiences mentioned by participants, including overnight hospital admissions or consultations with physicians, were self‐reported during interviews. No medical records or clinical verifications were obtained as part of this study. Therefore, any reference to hospitalization or medical consultation should be interpreted as subjective accounts rather than confirmed clinical events.

## 5 Conclusion

The catastrophic nature of war has a profound impact on nearly everyone, with older individuals being among the most vulnerable groups. Despite widespread awareness of its devastating effects on human life, war persists, and support efforts in response to it remain limited. Societies must unite to work toward the eradication of war. While it is essential to mitigate its devastating effects on all individuals, including the elderly, preventing war at all costs is of utmost importance. To mitigate the psychological and physical stress caused by war news on the elderly, it is essential to strengthen their resilience. This can be achieved by raising awareness on the subject, promoting stress control and management techniques, fostering social connections, and encouraging a proper understanding of the media environment. Most importantly, monitoring media content—such as implementing violent content labels to give the audience the right to choose—plays a crucial role in preventing the potential harm caused by media exposure.

## Author Contributions

Moatafa Moazam Fard and Milad Ahmadi Marzaleh were engaged in the study conception and design. All authors prepared the first draft of the manuscript. All authors did the analysis of the data and supervised the study. All authors have read and approved the final manuscript.

## Disclosure

The corresponding author (Mostafa Moazam Fard) affirms that this manuscript is an honest, accurate, and transparent account of the study being reported; that no important aspects of the study have been omitted; and that any discrepancies from the study as planned (and, if relevant, registered) have been explained.

## Ethics Statement

All ethical considerations were thoroughly addressed, and the study protocol was reviewed and approved by the Ethics Committee of Shiraz University of Medical Sciences. After obtaining the required permits from the Vice Chancellor of Research and the Faculty of Medical Management and Informatics, as well as an approval letter from Shiraz University of Medical Sciences, the researchers presented themselves to the participants and explained the study objectives and the research samples. Additionally, the Declaration of Helsinki was taken into consideration in this study.

## Consent

Participants were assured that all recorded information would remain confidential. Informed consent was obtained from all individual participants in the study. Subsequently, individuals who expressed their willingness to participate in the study were selected, and they were further reassured that they could withdraw at any stage of the interview process.

## Conflicts of Interest

The authors declare no conflicts of interest.

## Data Availability

The data sets used and analyzed during the current study are available from the corresponding author on reasonable request.
